# Emergency Department Utilization Among Adults With Intellectual and Developmental Disabilities: A Comparison of Those Using the Program for Adults With Intellectual and Developmental Disabilities and Those Using Alternative Primary Care Clinics

**DOI:** 10.7759/cureus.81667

**Published:** 2025-04-03

**Authors:** Jeremy M Williams, Kendyll Coxen, Carlos Palacio, Rafik Jacob

**Affiliations:** 1 Internal Medicine, University of Florida College of Medicine, Jacksonville, USA; 2 Emergency Medicine, University of Florida College of Medicine, Gainesville, USA; 3 Internal Medicine, Program for Adults With Intellectual and Developmental Disabilities, University of Florida College of Medicine, Jacksonville, USA

**Keywords:** adults with intellectual and developmental disabilities, emergency department utilization, healthcare policy, health care quality improvement, preventative care strategies, social determinants of health (sdoh), university of florida

## Abstract

Adults with intellectual and developmental disabilities (IDD) experience higher rates of emergency department (ED) utilization compared to the general population due to complex medical needs, healthcare access barriers, and social determinants of health. Although prior research has identified predictors of ED use, including socioeconomic status, primary care continuity, and housing stability, the role of specialized primary care programs in reducing reliance on emergency services remains understudied. This study examines the impact of the Program for Adults With Intellectual and Developmental Disabilities (PAIDD) at the University of Florida (UF) Health Jacksonville on ED utilization rates among adults with IDD. Using a retrospective cohort design, we compared ED visit frequencies between PAIDD patients and a control group of adults with IDD who receive care outside the program. Additionally, we explored whether housing status, living with family, independently, or in a facility, affects ED utilization. Findings revealed a statistically significant reduction in ED visits among PAIDD patients compared to the control group (p=0.004), suggesting that structured, multidisciplinary healthcare models improve continuity of care and reduce avoidable ED utilization. However, housing status alone did not emerge as an independent predictor of ED use (p=0.093), indicating that other factors, such as access to primary care and caregiver support, may play a greater role. These results underscore the need for expanded university-based IDD healthcare programs to bridge gaps in care, enhance preventive health strategies, and reduce emergency healthcare dependence in this population. The study supports the integration of dedicated IDD clinics in academic medical centers to improve long-term health outcomes and health equity for individuals with IDD.

## Introduction

Intellectual and developmental disabilities (IDD) is an umbrella term describing a group of neurodevelopmental disorders that cause lifetime impairment in cognition, communication, mobility, self-care, independent living, and adaptive behavior [1]. Studies found that individuals with IDDs typically have worse health outcomes. This is primarily due to limited access to proper health care services, polypharmacy, higher rates of poverty, malnutrition, and long periods of physical inactivity [2].

These social determinants of health, in combination with the medical complexity of many of these individuals, contribute to the fact that adult patients with IDD, on average, experience greater annual healthcare costs compared to those without IDD [3]. Inflation of direct costs is likely driven by increased emergency department (ED) utilization and increased use of other hospital-based services in this population. A recent study demonstrated that nearly 20% of ED visits among adults with intellectual disabilities in South Carolina were for an ambulatory care-sensitive condition, suggesting that timely provision of outpatient care could have prevented ED visits and/or inpatient hospitalization [4]. This study is meant to bring to light the utility of programs such as PAIDD and the positive financial impact it may have on healthcare utilization.

Program for Adults With Intellectual and Developmental Disabilities

The Program for Adults With Intellectual and Developmental Disabilities (PAIDD) at the University of Florida (UF) Health Jacksonville was established in 2013 as a continuation of the JaxHATS (Jacksonville Health and Transition Services) program. JaxHATS was designed to support the transition of care from pediatric to adult healthcare services for individuals with IDD, ensuring continuity in medical management as they aged out of pediatric care. Recognizing the ongoing need for dedicated adult care, Dr. David Wood, a pediatrician, and Dr. Linda Edwards, an internal medicine physician, co-founded PAIDD to provide a structured, specialized healthcare model for this underserved population. The PAIDD clinic was created in response to the significant barriers to healthcare access that adults with IDD often face, including difficulty finding providers trained in their unique medical and social needs. The program was designed to function as a comprehensive medical home, offering coordinated primary care and proactive health management to reduce unnecessary ED visits and hospitalizations. PAIDD emphasizes multidisciplinary, patient-centered care, collaborating with primary care physicians, specialists, and caregivers to develop tailored treatment plans. Regular screenings, proactive interventions, and health maintenance are essential for improving long-term outcomes in preventive and chronic disease management, while healthcare navigation support helps patients and caregivers access appropriate medical, social, and community resources. Since its inception, PAIDD has become a vital resource for adults with IDD, ensuring they receive consistent, specialized care in a setting designed to meet their complex health needs. By prioritizing preventive care and coordinated services, the clinic has helped lower ED utilization rates, improve health outcomes, and enhance patients' overall quality of life.

Housing status and its impact on emergency department utilization

Housing stability plays a critical role in healthcare access and outcomes for adults with IDD. Where an individual resides, whether in a family home, an independent living arrangement, or a specialized care facility, can influence their ability to receive consistent medical care and reduce reliance on emergency services [5]. Studies show that a large proportion of adults with IDD continue to live with family caregivers well into adulthood. McConkey et al. found that 85% of adults with IDD in an eight-year follow-up study remained in a family home, a trend that extends across different regions [6]. Chou and Schalock similarly reported that 92-95% of adults with IDD in Taiwan lived with family members, demonstrating a global reliance on familial support [7]. While family living environments often provide structured health monitoring and assistance with medical care, transitions to independent or group home settings can impact healthcare utilization patterns. For some individuals, moving into residential care facilities or independent living arrangements may reduce dependence on family for health management, leading to potential gaps in routine healthcare access [8]. However, structured residential programs may also provide better coordination of medical care than those in less stable housing situations. Research by Stancliffe et al. in the United States found that 42% of adults with IDD lived in non-family residential settings, highlighting a shift toward more independent or supported housing options [9]. This study analyzed housing status to determine whether living in different settings affects ED utilization rates.

## Materials and methods

Study design

This study employed a retrospective cohort design to evaluate the impact of the PAIDD clinic on ED utilization rates among adults aged 18 years and older with IDD. Participants consisted of adult patients with IDD who had utilized services within the UF Health Jacksonville ED system at least once during the five-year study timeframe; they were then divided into a control group and an experimental group based on the criteria below.

Study groups

The experimental group consisted of 107 adult patients with IDD who had been seen in the PAIDD clinic and had also visited the ED at least once within the study timeframe. The control group comprised 1,239 adult patients with IDD who had not been seen in the PAIDD clinic but had received medical care at the UF Health Jacksonville ED system within the five-year timeframe. Individuals aged 90 years or older and those seen at the PAIDD clinic were excluded from the control group.

Time frame

The evaluation period for the study groups encompassed individuals who visited the UF Health Jacksonville ED system between March 1, 2019, and March 1, 2024. For the experimental group, patients must have also received care at the PAIDD clinic at least once during this time.

Data management and coding

Data was obtained retrospectively through the Integrated Data Repository (IDR) at the UF. The study team received a list of patients from the IDR who met the inclusion criteria for each group. Patient charts were accessed to determine their living situation; whether in a facility, family home, or lived independently. Data management involves coding to maintain confidentiality. Once the original data was collected and the chart review was completed, identifiers were removed, and each subject was assigned a unique research identification number. Dates pertaining to medical events were converted to relevant time frames, such as time to discharge or time to readmission. All coded data was securely stored in an encrypted, password-protected Excel file located on a secure server within the UF College of Medicine. Access to this data was restricted to the study team members only.

Data analysis plan

The data analysis included the use of descriptive statistics to derive rates of ED utilization for each group. A comparative analysis between the two groups was conducted using an independent sample t-test to analyze the primary endpoint. Both groups were further divided into subgroups based on housing status. An analysis of variance (ANOVA) test was performed on each group to determine if housing status was an independent predictor of ED utilization between subgroups. A significance level of p<0.05 was used to determine statistical significance. Once the data was collected, identities were de-identified, ensuring that only coded data was utilized for analysis. The coded data was stored securely, and the coded list identifying participants was destroyed upon study completion. All keys were deleted to maintain data integrity.

## Results

This study utilized retrospective data on adult patients with IDD obtained through the IDR at the UF. It included a total of 1,518 patients over a five-year period between March 1, 2019, and March 1, 2024. Participants were divided into two distinct groups: the control group (n=1,239) included patients who were seen in the ED but not PAIDD clinic; the experimental group (n=107) included patients who were seen in the ED and also seen in the PAIDD clinic. Each group was then subdivided based on whether they resided in a family home, lived independently, or in a specialized care facility. A significance level of p<0.05 was established to determine any significant differences in ED utilization rates.

A summary of the demographics of patients included in the study is shown in Table 1, which is subdivided into groups based on both sex and housing status. As seen in Table 1, both study groups comprised a majority of males; the control group had 742 (60%), and the experimental group had 61 (57%). Concerning housing status, the control group had roughly the same amount of participants from both family homes or living independently, 513 (41%) and 523 (42%), respectively, while only 203 (17%) resided in an assisted living facility. Similarly to the control, the experimental group had the least representation from participants residing in assisted living facilities, 5 (5%); however, there were notably more patients from family homes, 73 (68%), than those who lived independently, 29 (27%). 

**Table 1:** Demographics and Clinical Characteristics of Patient Population Demographic data for both control and experimental groups divided by sex and housing status.

Characteristic	Control (N=1,239)	Experimental (N=107)
Age (years)	39.9	32.3
Sex		
Male	742 (60%)	61 (57%)
Female	497 (40%)	46 (43%)
Housing status		
Family home	513 (41%)	73 (68%)
Independent	523 (42%)	29 (27%)
Facility	203 (17%)	5 (5%)

Table 2 depicts outcomes for both primary endpoint and secondary analysis. The primary endpoint compared the average number of ED visits during the five-year study period between the control group and experimental group using a paired t-test. Our analysis revealed the average number of ED visits was statistically higher in the control group (6.40, SD (10.34)) than in the experimental group (3.71, SD (3.94)); P=0.004, t-statistic=-7.062.

**Table 2:** Primary Endpoint and Secondary Analysis ED visits (total # (average)) over the study timeframe for both control (n=1,239) and experimental (n=107) groups with associated statistical analysis. Primary endpoint: comparison of average ED visits based on PAIDD status determined by paired t-test. Secondary analysis: comparison of average ED visits within both control and experimental groups based on housing status alone determined by one-way ANOVA. ED, emergency department; PAIDD, Program for Adults With Intellectual and Developmental Disabilities

	PAIDD status (primary endpoint)		Housing status (secondary analysis)	
	Control	Experimental	Control	Experimental
Family home	-	-	3,180 (6.12)	265 (3.63)
Independent	-	-	3,444 (6.59)	37 (7.40)
Facility	-	-	1,310 (6.45)	96 (3.31)
Total	7,862 (6.40)	398 (3.71)	-	-
Test statistic	-7.062 (T-statistic)		0.183 (F-statistic)	2.426 (F-statistic)
P-value	0.004		0.833	0.093

For the secondary analysis, the control group and experimental group were subdivided based on housing status: control (family home (n=513), independent (n=523), facility (n=203)) and experimental groups (family home (n=73), independent (n=29), facility (n=5)). Using one-way ANOVA, no statistically significant difference was found in the average number of ED visits based on housing status alone within either the control group (P=0.833, F-statistic=0.183) or the experimental group (P=0.093, F-statistic=2.426).

## Discussion

Adults with IDD often face systemic barriers to healthcare, including limited provider knowledge, difficulty accessing primary care, and higher rates of chronic conditions like diabetes and obesity [10]. Programs like PAIDD help bridge these gaps by offering comprehensive, coordinated care tailored to individuals with IDD. This study demonstrates a statistically significant reduction in ED utilization among adults with IDD who receive care at PAIDD compared to those who do not. These findings align with existing research, such as Wood, et al., that emphasizes the importance of specialized primary care in reducing ED visits for this population [11]. Studies have shown that appropriate primary care and model care programs focusing on prevention and integrated support can lower ED use for those with IDD, which supports the results of this study. The statistically significant reduction in ED visits among PAIDD patients suggests that specialized primary care models can effectively mitigate preventable ED utilization and improve health outcomes by improving the continuity of primary care and helping to prioritize preventative measures.

Housing status and emergency department utilization

While previous research, as noted by Friedman [12], identifies housing status as a social determinant of health, wherein individuals with more stable housing generally have improved access to medical care and better health outcomes, our analysis did not identify a statistically significant difference in ED utilization by housing status alone. These results indicate that other factors like routine utilization of primary care services, caregiver support, or individual health needs may play a more significant role in influencing ED utilization for adults with IDD. The World Health Organization recognizes social determinants of health as a large contributor to overall inequity in health outcomes. In particular, social determinants such as poverty and education, specifically for individuals with disabilities, heavily influence health outcomes [13]. These findings highlight the need for further research into the interaction between housing status, social determinants of health, and ED use among adults with IDD.

Implications for healthcare policy and practice

Other studies have identified obstacles to healthcare for people with IDD, including provider training, communication deficits, lack of knowledge, and caregiver support. These barriers may explain why adults with IDD often experience higher rates of ED utilization compared to the general population [14]. The results of this study reinforce the need for integrated healthcare models that prioritize person-centered, coordinated care to prevent unnecessary ED visits. PAIDD addresses these challenges by functioning as a comprehensive medical home. This is achieved through multidisciplinary collaboration, enhanced provider education and training, and access to routine primary care tailored to individuals with IDD. Furthermore, similar programs (i.e., HealthMatters) have shown that a structured approach improves health outcomes among individuals with IDD, which supports the argument that health management is improved through interventions like PAIDD [15].

Several limitations should be considered when interpreting these results. The sample size of the experimental group (n=107) was significantly lower compared to the control group (n=1,239), which may reduce the external validity of this study compared to that of the general population. This is secondary to the limited number of patients routinely seen in the PAIDD clinic available within the UF IDR. Furthermore, the social determinants of health among this patient population may differ in relation to geographic location, which may affect regional ED utilization rates among patients with IDD. Confounding bias is an additional possibility; this would be due to group differences such as age or the exclusion of those who avoided ED care altogether due to effective outpatient services. Finally, as a retrospective cohort study, this research is subject to potential recall bias, selection bias, or confounding. These limitations highlight the need for future research utilizing larger study groups within various geographical regions to improve external validity. Additionally, researchers should explore longitudinal ED utilization trends among PAIDD patients to assess the impact of specific interventions, such as care coordination programs and telemedicine services, in reducing emergency care reliance.

## Conclusions

This study highlights the significant impact of specialized primary care services, such as PAIDD, in reducing ED utilization among adults with IDD. The findings emphasize the importance of dedicated, multidisciplinary healthcare models in addressing this population's complex medical and social needs. PAIDD helps mitigate systemic barriers to healthcare access and reduces unnecessary reliance on emergency services by providing consistent, coordinated care. While housing status did not emerge as a statistically significant predictor of ED utilization in this study, this finding suggests that other social and healthcare-related factors, such as continuity of primary care, caregiver involvement, and systemic healthcare accessibility, may play a more critical role in determining ED use among adults with IDD. Future research should explore the long-term impact of specialized programs like PAIDD on preventive care engagement, health outcomes, and healthcare cost reduction. Given the ongoing healthcare disparities faced by adults with IDD, there is a strong need for the expansion of university-based programs like PAIDD. Similar programs at other academic medical centers could help bridge gaps in care, train future healthcare professionals in IDD-specific care, and provide a structured model for comprehensive, long-term management of IDD patients. By integrating education, clinical care, and research, university-based programs can drive systemic change, improve health equity, and reduce healthcare disparities for individuals with IDD across different regions. The results underscore the urgent need for healthcare policies that support the development of such programs, ensuring that adults with IDD receive the specialized care they need to lead healthier, more independent lives.
